# Model-based risk assessment and public health analysis to prevent Lyme disease

**DOI:** 10.1098/rsos.170841

**Published:** 2017-11-15

**Authors:** Nasser Sharareh, Nasim S. Sabounchi, Amanda Roome, Rita Spathis, Ralph M. Garruto

**Affiliations:** 1 Systems Science and Industrial Engineering Department, The State University of New York at Binghamton, Binghamton, NY, USA; 2 Anthropology Department, The State University of New York at Binghamton, Binghamton, NY, USA; 3 Laboratory of Biomedical Anthropology and Neurosciences, Anthropology Department, The State University of New York at Binghamton, Binghamton, NY, USA; 4 Biological Sciences Department, The State University of New York at Binghamton, Binghamton, NY, USA

**Keywords:** system dynamics modelling, human behaviour, environmental risk, vector, reservoir, Lyme disease

## Abstract

The number of Lyme disease (LD) cases in the northeastern United States has been dramatically increasing with over 300 000 new cases each year. This is due to numerous factors interacting over time including low public awareness of LD, risk behaviours and clothing choices, ecological and climatic factors, an increase in rodents within ecologically fragmented peri-urban built environments and an increase in tick density and infectivity in such environments. We have used a system dynamics (SD) approach to develop a simulation tool to evaluate the significance of risk factors in replicating historical trends of LD cases, and to investigate the influence of different interventions, such as increasing awareness, controlling clothing risk and reducing mouse populations, in reducing LD risk. The model accurately replicates historical trends of LD cases. Among several interventions tested using the simulation model, increasing public awareness most significantly reduces the number of LD cases. This model provides recommendations for LD prevention, including further educational programmes to raise awareness and control behavioural risk. This model has the potential to be used by the public health community to assess the risk of exposure to LD.

## 1. Introduction and background

Lyme disease (LD) is a bacterial infection caused by *Borrelia burgdorferi*, which is transmitted to humans through the bite of an infected blacklegged tick (*Ixodes scapularis*), also known as the deer tick [1], and can lead to serious neurologic and cardiac complications if not properly treated. It is among the top 10 most prevalent infectious diseases [2], and the most common vector-borne disease in the USA [3], with over 300 000 new cases diagnosed each year based on data reported by CDC [4]. Recent reports have suggested that 36% of patients diagnosed with LD go on to develop post-treatment LD syndrome (PTLDS), in which patients have persistent symptoms for months to years after diagnosis and treatment [5]. In addition, in the USA the corresponding medical cost of LD and PTLDS has a strong negative economic impact and can cost between $712 million and $1.3 billion each year [6]. It is important to note that some factors like the lack of healthcare provider's knowledge, non-specific symptoms and unrecognized tick bites may delay on-time diagnosis of LD, leading to the chronic stage of PTLDS [7].

Data for LD cases in different states throughout the US show that there is a large variation in the number of cases geographically [8]. Several factors may influence variation in incidence rates of human LD throughout the USA [9], such as differences in climate, prevention strategies within each state, awareness of the public and recognition of symptoms by physicians and human exposure to ticks. In addition, LD cases are strongly correlated with the density of infected nymphs which varies by state [9].

In prior modelling work on LD, the influence of various factors including temperature and seasonal variation and associated risk on LD incidence have been modelled. For instance, an agent-based model developed for Scotland shows that higher temperature influences the tick population by affecting behaviour, fertility and survival rate and habitat suitability of hosts, which could contribute to a higher tick–host contact frequency and pathogen transmission [10]. Another study reflects how an increase in global temperature is leading to an increase in tick habitat [11]. Other simulation studies have focused on different kinds of tick hosts and their effect on tick populations. A mathematical model was developed to examine risk of LD with different densities of reservoir hosts, such as rodents and tick predators like songbirds in different regions in Dutchess County located in the Hudson River Valley of New York State [12]. The authors found that songbirds have a low impact on reducing the prevalence of tick infection, especially when there is a high density of rodents.

Finally, environmental and public activities such as the disturbance of wooded areas and forests lead to an increase in populations of white-footed mice, the competent reservoir host in the US and an increase in risk of human exposure to ticks; also, the lack of predation and an increase in ecologically fragmented peri-urban built environment areas will increase mouse populations [13].

While some researchers have, in general, studied the factors involved in contracting LD, others have demonstrated the risk of contracting LD in a specific geographical area. For example, Kugeler *et al*. [8] defined a method to determine high-risk counties for LD based on observed and reported LD cases and Vourc'h *et al.* [14] provide a snapshot map of the risk of contracting LD in a forest in France and analysed different factors influencing risk. Mapping risk of exposure to the LD infectious agent in a specific geographical area will help focus on region-specific prevention measures and increase awareness by the public to avoid tick-infested places [14]. Additionally, computer simulation has been used to determine the influence of controlling hosts at different tick stages on tick populations and found that controlling hosts for larvae and nymphs had the largest effect on controlling the tick population [12]. The authors mention that it is not practical to use their model to predict LD dynamics for different regions because there is not enough data for host density in particular locations.

Overall, most studies focused only on a limited number of factors associated with LD risk because the current understanding of transmission of LD to human populations primarily depends on the presence of infected ticks and various ecological variables [15–17]. Human behaviour and demographic factors governing the risk of transmission have not been primarily considered in studying risk of infection for LD. Therefore, an in-depth study of the role of human behaviour in the spread of LD remains a critical gap in our understanding, mitigation and prevention of human infection [18].

In this study, we use a system dynamics (SD) approach to understand LD spread and include human behavioural and demographic factors in our model. In an SD approach, we focus on understanding the relationship between the structure of a system and the resulting dynamic behaviours generated through multiple interacting feedback loops [19]. Owing to the multiple ecological, demographic and behavioural risk factors interacting over time, LD transmission is a complex problem, and an SD approach is an optimal choice for modelling LD transmission dynamics.

A few studies have applied SD for LD modelling [20,21]. One study investigated the influence of awareness on LD cases and patient outcomes, and concludes that public-based interventions which focus solely on increasing public awareness have a higher impact than physician-based interventions on decreasing LD cases [20]. Also, SD modelling was used to replicate a Dutch National Risk Assessment (NRA) approach to investigate the risk of LD and found that human behavioural modification that focuses on practices to prevent tick bites and infection after being bitten are considered cost-effective and universally applicable [21]. The study concludes that public-based interventions have a higher impact than physician-based interventions on decreasing LD cases [21].

As a result of our interdisciplinary research collaboration between Systems Science and Industrial Engineering and Anthropology, the SD modelling framework is the first comprehensive modelling research for Lyme disease that captures different behavioural and environmental risk factors including public awareness, tick density, rodent density, environmental risk, seasonality, human risk and their interactions to capture LD transmission to humans. The model formulates and captures soft variables such as awareness about LD and risky behaviour of individuals. The simulation model is refined based on significantly detailed data that have been collected ecologically, molecularly, demographically and behaviourally over a 2-year period, 2013 and 2014. The simulation model result for the number of LD cases is validated against the time-series data available for the number of LD cases within Broome County, NY [22]. The data show an increasing trend from 5 LD cases in 2000 to 201 cases in 2015.

The purpose of the current research is to develop a simulation framework that illustrates risk factors and the interactions that are driving the increase in LD cases. For our model-based assessment, the main geographical area of focus is the State University of New York at Binghamton campus, a 930-acre campus with a student, faculty and staff population of more than 18 000 individuals.

## 2. Methodology and model development

In order to develop the SD model, there are several steps involved, beginning with defining the dynamic problem, and then illustrating a causal map that consists of factors that interact with each other in a linear or nonlinear fashion, which creates the system's behaviour. Next, the simulation model is developed by converting this conceptual map to an analytical tool, and lastly, after validating the model, it is tested in various intervention scenarios to evaluate their outcomes for designing effective policies, programmes or mitigation strategies [19]. The full documentation of this model is available in the electronic supplementary materials.

**Figure 1.**
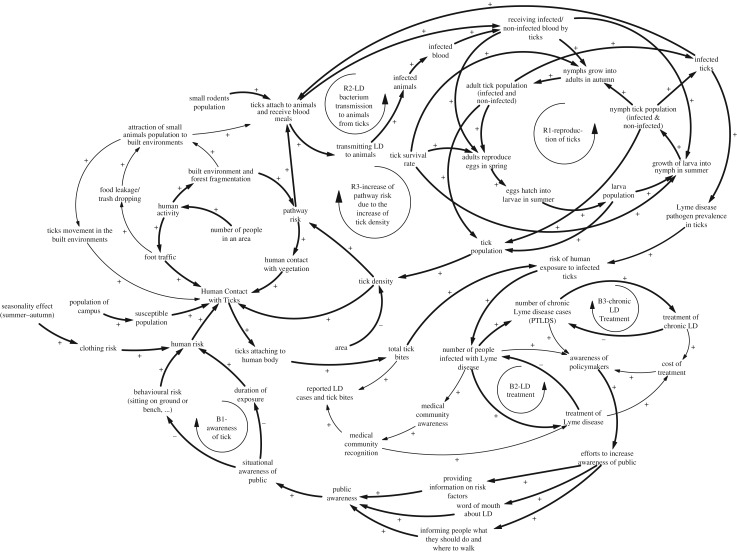
Causal loop diagram.

In this paper, the dynamic problem of the study is the increasing trend of LD cases. The underlying causal factors and feedback structure that have led to the continuing growth of LD incidence are described in a causal loop diagram in figure 1. We use this conceptual diagram to develop our dynamics hypothesis based on the significant factors that drive the high number of LD cases. Later, considering the feasibility and availability of data to validate the structure, we simulate segments of the qualitative model that are bolded in figure 1 for quantitative analysis of the risk factors for LD. Finally, we use the simulation model to test various intervention scenarios and their impact on reducing the number of LD cases.

### 2.1. Causal loop diagram

The causal loop diagram consists of causal links that form the feedback loops. The purpose of this diagram is to frame our hypothesis that explains the dynamic trend of increase in LD cases as presented in figure 3. Hence the causal links are not verified until we simulate the model and further analyse the validity of this diagram. We draw the causal links by reviewing the literature and talking with researchers in the LD field.

As outlined in the causal loop diagram (figure 1), and based on our preliminary research over a 2-year period on the Binghamton University (BU) campus [23,24] including an online survey administered in 2014, as well as from the literature [2,6–10,12,14,17,20,25–38], our hypothesis is that the prevalent reinforcing and balancing feedback loops lead to an increase in LD cases since the year 2000. The arrows in figure 1 illustrate causalities among variables that can have a positive or negative sign; positive means that changes in one variable lead to the changes in another variable in the same direction, while negative leads to changes in opposite directions. All this information comes from the literature review, talking to researchers in the LD field, and our basic knowledge about the system. In this diagram, the bold arrows are the ones that have been converted to the simulation model, meaning that numerical and differential equations have been defined for them. The reason that we chose just a subset of them was based on the available data and information. Also, for simplification purposes, we did not consider the influence of food leakage and awareness of policymakers on the system.

As shown in the causal loop diagram (figure 1), several factors will increase human contact with ticks, including an increase in the campus population, growth of the built environment, walkway risk and human risk. With regard to walkway risk, two different types of walkways have been considered; organic (dirt or grass) and non-organic (asphalt or concrete) of which the former has a higher risk. With regard to human risk, behavioural risk factors such as sitting on grass, walking through woodchips, sitting on the ground or at a picnic table and clothing risk are considered.

An increase in human contact with ticks will increase the probability of infected tick bites, and therefore, an increase in the number of LD cases. The growth of LD cases will attract the attention of public health officials and raise the medical community awareness of the disease. If the medical community's awareness is increasing, the recognition and diagnosis of LD cases will increase. Likewise, increased awareness by public health officials and policymakers leads to interventions to increase public awareness; these interventions can be implemented through focus groups or advocacy groups by providing information on LD and risk factors. Once the public is more aware, behavioural risk and exposure of humans to infected ticks decreases, leading to a subsequent drop in LD cases (Loop B1). The detailed components of each loop are mentioned in table 1.

**Table 1.** Loops elements.

loop name	components
R1	eggs hatch into larvae in summer → larva population → growth of larva into nymph in summer → nymph tick population (infected and non-infected) → nymphs grow into adults in autumn → adult tick population (infected and non-infected) → adults reproduce eggs in spring → eggs hatch into larvae in summer
R2	infected ticks → ticks attach to animals and receive blood meals → transmitting LD to animals → infected animals → infected blood → receiving infected blood by ticks → growth of different life cycle stages of ticks → infected ticks
R3	pathway risk → ticks attach to animals and receive blood meals → receiving non-infected blood by ticks → growth of different life cycle stages of ticks → tick population → tick density → pathway risk
B1	humans in contact with ticks → ticks attaching to human body → total tick bites → risk of human exposure to infected ticks → number of people infected with Lyme disease → awareness of policymakers → efforts to increase awareness of public → public awareness → situational awareness of public → duration of exposure/behavioural risk (sitting on ground or bench, …) → human risk → human contact with ticks
B2	number of people infected with Lyme disease → treatment of Lyme disease → number of people infected with Lyme disease
B3	number of chronic Lyme disease cases (PTLDS) → treatment of chronic LD → number of chronic Lyme disease cases (PTLDS)

The most important risk factors in the growth of LD cases are the density and infectivity of the tick vector (*Ixodes scapularis*) population as well as the competent rodent reservoir (*Peromyscus leucopus*) population. The tick's life cycle starts with an egg, which hatches into a larva, and then moults into a nymph and finally moults again and becomes an adult (Loop R1). During each stage, from larva through adult, ticks will have an estimated probability of survival, and at each stage, larva through adult have the potential to become infected depending on the availability of a blood meal from an animal infected with the LD pathogen, *Borrelia burgdorferi*.

The attraction of small animals, such as mice, within ecologically fragmented peri-urban built environments and growth of the rodent population leads to more hosts and more blood meals for ticks [39]. Furthermore, ticks have the ability to become mobile by attaching to rodents or other animals, such as white tailed deer. After an infected tick attaches to a host for a blood meal, the LD bacterium can be transmitted from the saliva of the infected tick to the host, including humans as dead end hosts. Consequently, an increase in tick infectivity will increase the prevalence of infected animals exponentially, depicted in the reinforcing loop R2. In addition, the increase in infected ticks will increase the walkway risk because more rodents and humans are likely to come in contact with these ticks (Loop R3); therefore, there will be more LD and potentially more cases of PTLDS. However, with increasing recognition of tick bites and non-specific symptoms and awareness of LD, the medical community can decrease these numbers (Loop B2 and B3).

### 2.2. Field methods

Data were collected on LD and associated risk factors that include tick and rodent density and infectivity, and environmental and human behavioural and demographic risk over the course of two consecutive years, 2013 and 2014 from the BU campus in the Southern Tier of New York State. Two residential dormitory sites (Hillside and Susquehanna) and the campus' Nature Preserve trail system that is over 182 acres, were used to collect these data. Each walkway length was measured in metres using an open reel measuring tape to calculate tick and rodent density.

These areas were chosen to represent ecologically fragmented peri-urban built environments [40], where people are regularly perambulating or engaging in other regular activities, thus providing ideal settings for the potential transmission of tick-borne diseases. The conventional assumption is that built environments offer minimal risk for contact and subsequent infection from ticks. Consequently, precautions typically taken in non-built, more remote or wilderness areas are probably not taken in built environments. Additionally, ticks are attracted to CO_2_ and thermal outputs given off by humans and other animals [41], potentially increasing their risk of contact and LD infection.

Ticks and rodents were collected within 3 m of each side of walkways of high human use at each of the survey sites. Ticks were collected from April 2013 to November 2014 excluding winter (December through March) using the traditional dragging method described by researchers and health professionals, yielding 1254 ticks [42]. Briefly, 1 m^2^ corduroy cloths were dragged over low-lying vegetation to collect *I. scapularis* ticks, the primary vector for *B. burgdorferi*. Questing ticks (in search of a blood meal) were removed from the cloths with forceps, placed into sterile cryovials containing 70% ethanol and were transported to the laboratory for pathogen analysis. The total area dragged for ticks around all 22 walkways was 71 534 m^2^ (7.1 ha). Data were recorded on data sheets to note the walkway, number of ticks collected and the area dragged so accurate tick densities could be calculated.

*Ixodes scapularis* species were identified microscopically and life cycle stage and sex were determined, and catalogued by location. Ticks were flash frozen in liquid nitrogen, physically disrupted using chrome steel beads with a TissueLyser LT bead mill and DNA extracted with the Qiagen DNeasy Blood & Tissue Kit according to the manufacturer's instructions. The presence of *B. burgdorferi* was assessed using *ospC* pathogen-specific primers [43]. PCR amplification products were assessed using gel electrophoresis and amplicons were subjected to Sanger sequencing to confirm pathogen identity.

From September to November 2014, 112 rodents were collected within 3 m of each side of the same walkways from which ticks were collected, using approved Institutional Animal Care Use Committee (IACUC) protocols (#703-12 and #746-15). Sherman live traps were set in the evening, baited with peanut butter and oats, and checked the following morning. *Peromyscus leucopus,* the primary reservoir for *B. burgdorferi* was identified in the field, and *P. leucopus* density estimated according to standard rodent densities per acre [44] and converted to hectares. Rodents were sacrificed according to the American Veterinarian Medical Association's guidelines [45], and transported to the laboratory where their tissues were harvested and tested for *B. burgdorferi.*

Demographic and behavioural data were compiled from observations that took place in 30 min increments over an 11 h time period (08.00–19.00 h) for three days (two weekdays and one weekend day) on each walkway where ticks and mice are collected. Walkway composition and location were recorded, along with weather, the presence of trash cans/dumpsters and their contents. The number of individuals that were recorded on a data sheet for gender, skin exposure (noting what part of the body) and behavioural risk (walking through grass, sitting on a bench and sitting on the ground) was 11 419. These data were then compiled and 9354 risk events determined. Risk events are defined as an event where behavioural risk (sitting on grass, walking across woodchips, etc.) or clothing risk (i.e. skin exposure) is observed. Events may represent the same individual more than once as they walk to and from areas on the BU campus.

Using anthropological, ecological and mathematical approaches, these comprehensive variables can be modelled to predict the risk of disease transmission with better resolution and specificity to particular environments where the fragmented peri-urban built environment is the predominant ecological feature.

### 2.3. Building the simulation model

Building upon the causal structure diagram in figure 1, we have developed an SD simulation model to evaluate significance of different risk factors in replicating the historical trend for the number of LD cases in all 22 observed walkways; among them, 11 organic and 11 non-organic (asphalt or concrete), at the BU campus in the Southern Tier of New York. We used differential equations to develop the simulation model. Figure 1 is an illustration of factors and the underlying structure of the system that yield the final behaviour of the system. However, in order to simulate the model, we have to convert this conceptual model to an analytical model to investigate ‘what–if' strategies and run different simulation runs. Also, SD is a deterministic approach but we consider uncertainty through policy analysis that we have shown in the results section. Each walkway has its own susceptible human population, tick density, walkway risk and human risk that were determination based on the methods discussed earlier. Once these factors were determined, we simulated the number of LD cases that occurred between 2008 and 2015 for all 22 walkways (see Calibration section).

The model provides a framework for evaluating the impact of various environmental, demographic, behavioural, vector and reservoir risk factors for LD spread and recommendations for the need to develop further preventive strategies and to control tick population growth.

**Figure 2.**
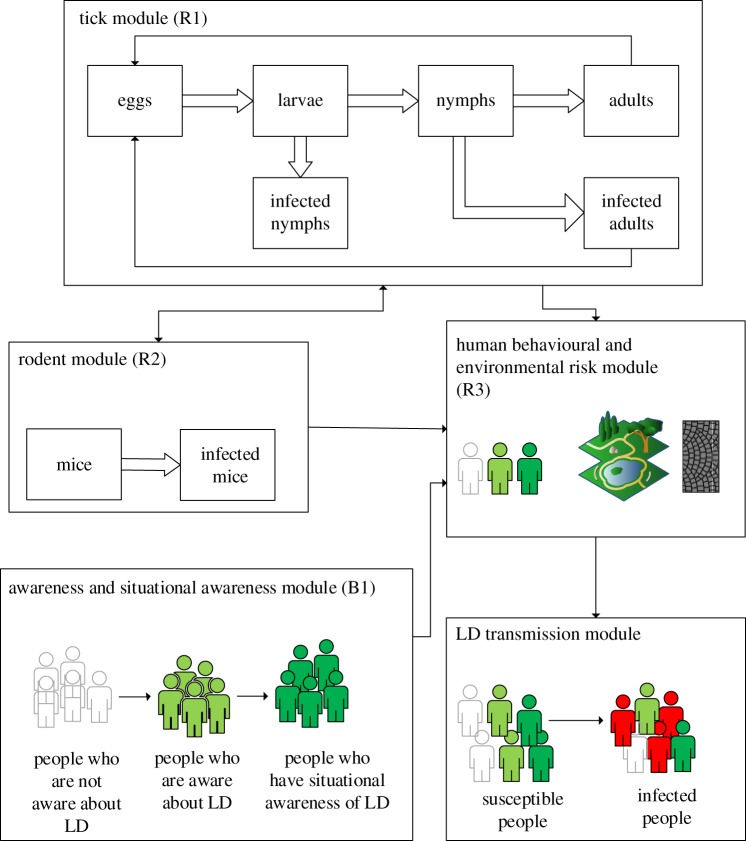
Schematic view of simulation model.

The model consists of five different modules. The modules and interactions among them are illustrated in figure 2.

#### 2.3.1. Tick cycle module

The tick cycle module is the comprehensive section of the model that simulates the tick population by capturing different life cycle stages of tick populations (larva, nymph and adult) (Loop R1 in figure 1), adding the seasonal factors to the model (different rate of mating of adults in autumn and spring) and capturing the density of infected ticks through the infectious transmission loop (Loop R2 in figure 1) between ticks and animals. Ticks will survive each stage of their life cycle, with an estimated probability, and at the larval stage can become infected depending on the availability of infected blood meals from host animals. The reproduction cycle of ticks typically takes approximately 2 or 3 years and factors such as the habitat and the existence of blood meals can facilitate the growth of tick populations. In the northeastern USA, existence of ticks at each stage of the life cycle is limited throughout the year. Except for larvae that can exist during all seasons, adults are found only during the autumn, winter and spring, while nymphs are found during late spring, summer and autumn seasons, and eggs only in the spring and summer months [46].

The simulation results of this module are compared with the tick density and density of infected ticks for 2013 and 2014. The comparison is made using the maximum-likelihood estimation approach and calibrating the simulation model against the historical data series discussed in the Results section. Since there are a different number of samples for each walkway, and each sample has a different number of ticks, thus, a mean trimmed 20% has been used to calculate the average number of ticks for each walkway with the outliers removed in the sample data. Since the sample number of ticks that are collected by dragging corduroy cloths over the walkway area, accounts for only 6.3% of all the ticks within that walkway area [47], we estimate the total population of ticks for each walkway, by multiplying the average number of ticks from the collected samples, by 15.87 (i.e. the inverse of 6.3%). In addition, based on the infectivity level of collected ticks that has been determined molecularly in our laboratory, the number of infected ticks on each walkway is calculated.

#### 2.3.2. Rodent module

In this module, rodents only include *P. leucopus*, the most competent reservoir host for *B. burgdorferi*; however, other rodent species can be included if there are data available for these less competent hosts. Currently, the reproduction, death and infection rate of *P. leucopus,* the white-footed mouse, have been modelled. The number of mice in the area was estimated based on the reproduction and longevity information from Aguilar [44]. A mouse becomes infected when an infected tick attaches and takes a blood meal, and an infected mouse can infect ticks that feed on it. Since the home range for mice is on average about 7 m [48], and also for simplification purposes, we assume that a mouse within a walkway will not move to other walkways, as walkways are on average, much more than 7 m apart. A mouse has as estimated likelihood of being bitten by an infected tick in a walkway area (walkway plus 3 m on each side of it) based on the density of ticks, number of infected ticks and the number of mice in that walkway area. As each of these factors increases, the mice on a given walkway have a higher risk of becoming infected. Thus, the infection rate of mice on a walkway is calculated based on input from the tick module, including density of ticks and number of infected ticks.

#### 2.3.3. Human behavioural and environmental risk module

In this module, we have human behavioural risk which is estimated based upon clothing and behavioural risk of individuals, and environmental risk. The former is estimated using observational data collected on the BU campus in 2013 and 2014. Data on the number of risky events, such as lying on the grass or not fully covering the body and thus helping to protect against tick bites, has been recorded by research team observers among individuals walking along each walkway; in an 11-hour day, 9354 risk events were observed on all 22 walkways. However, because observational data on human behaviours were gathered only during the autumn of 2013 and spring of 2014, the estimated risk does not represent the real risk for all seasons. The risk would be lower during colder periods including winter and most of the spring season on the campus in Binghamton, NY. Additionally, during the summer, only a very small number of students are in attendance. Therefore, the clothing and activity risk fluctuates due to weather conditions and seasonality. The same risk estimate for each walkway from the observational data is used for summer and autumn seasons and is decreased by a fraction during other seasons.

The environmental risk has been captured based on walkway type and the corresponding tick density on each walkway that is calculated from the tick module. The directed arrow that links the tick module to the human behavioural and environmental module in figure 2 shows this relationship. As a result, when the tick density is high and the walkway is an organic walkway (dirt, grass or woodchips), then the environmental risk is much higher than on a non-organic walkway (asphalt or concrete) with the same tick density.

#### 2.3.4. Awareness and situational awareness module

This module models how awareness and situational awareness is changing over time and influencing the behavioural risk of contracting LD. Awareness is defined as basic knowledge about ticks and LD. It does not directly affect the behavioural risk of individuals in protecting themselves against LD. When awareness increases, it leads to the gradual growth of the situational awareness of people. The model captures this behaviour, by a second-order delay structure.

Situational awareness is defined as the understanding of the situation and helps individuals to lower their behavioural risk, for example, to know where to walk and sit outdoors to lessen the risk of tick bites and how to remove a tick if they observe one attached to their body to reduce the risk of contracting the disease. In this model we assume that once individuals acquire situational awareness, they will retain it. According to Liao *et al.* [49], situational awareness is a combination of self-efficacy, perceived susceptibility and understanding of the situation, and this concept has been identified during World War I to express how important it is to gain awareness about a situation before your enemy gets to that same point of awareness [32]. Salmon *et al.* [50] gives a very useful summary of situational awareness, its relevance to complex systems and how to measure it.

As situational awareness increases, behavioural risk will decline and consequently the likelihood of getting LD from an infected bite will decrease. This explains the connection between the ‘awareness and situational awareness’ module and the ‘human behavioural and environmental risk' module (see figure 2).

#### 2.3.5. Lyme disease transmission module

The final module captures LD transmission by using a basic susceptible–infected–recovered structure whereby people contract LD by being bitten by infected ticks, and can recover if they are treated, following which recovered people again rejoin the susceptibility pool. Based upon the total risk for LD estimated within the ‘human and environmental module', the rate of susceptible people who walk along the 22 walkways on the campus on a daily basis and become infected from an infected tick is simulated. This rate will generate the number of LD cases on each walkway, and then sum the total cases for all 22 walkways on the campus compared to the historical data on cases of LD.

### 2.4. Calibration

The parameters in the simulation model have been determined using (i) the ecological and behavioural and demographic observational data that we have collected and discussed in the ‘Field methods' section, (ii) the published literature and reports [3,44,46,51–57], and (iii) calibrating the model towards historical trends for the number of LD cases that occurred on the University campus (see below), if other sources do not exist. We calibrated the simulation model against the historical data series from table 2 and two more time-series data on tick densities, using the maximum-likelihood estimation approach. During calibration, the sum of weighted differences between the time-series data of ‘LD Cases', ‘tick density' and ‘infected tick density' and their respective simulated values are minimized.

**Table 2.** Scaling the Broome County data from New York State Department of Health to the BU campus level.

	2008	2009	2010	2011	2012	2013	2014	2015
reported Broome County LD cases [22]	19	24	22	62	64	207	152	201
adjusted Broome County LD cases by 20% increase [60]	22.8	28.8	26.4	74.4	76.8	248.4	182.4	241.2
Broome County population [59]	194 635	194 630	200 428	199 335	198 670	198 203	197 349	196 567
Campus population (students and faculty members) [58]	15 776	15 584	15 741	15 594	16 174	16 985	17 639	17 891
scaled down Broome County LD cases to campus size	1.84	2.3	2.07	5.82	6.25	21.28	16.3	21.95

To compare the output of the simulation model with the historical data, we need the number of LD cases that occurred on the BU campus. The number of LD cases is only available for Broome County where BU is located. Thus, the Broome County data were downloaded from the New York State Department of Health [22] and then scaled down according to the ratio of the campus population [58] to the Broome County population [59]. However, according to White *et al.* [60], the reported LD cases are not the actual number and the percentage of cases that are underreported is at least 20%. Some reasons for underreporting are described as a lack of provider's knowledge and also case misclassification [60]. Therefore, we increased the number of reported LD cases after 2008 by 20% (see table 2). We chose 2008 as our initial time for simulation due to the change in the case definition of LD.

The final parameter values and corresponding references are reported in table 3. Most parameters have the same value for all walkways on the campus, such as average infection time or average time between different stages of the tick's life cycle, but some have different values from one walkway to another; for example, the available infected blood meals from rodents for each walkway area indicates the available infected rodents for each walkway. The upper part of table 3 contains the parameters that we could find reliable intervals for, and the lower part includes the ones in which values were calibrated. In addition, although we used literature and researchers in the LD field to define some intervals, most of the values reported in the literature were estimations; thus, we decided to choose the values that make more sense based on our observations. We also tried to define the average time interval of tick life stages in a way that produces a 2-year tick life cycle. That explains why some of the parameters in the upper part of table 3 may be outside the recommended intervals from the literature (these variables are labelled with *).

**Table 3.** Parameter's values (from literature or by calibration) and references.

parameters	literature value [references]	we used
larva gets blood and is inactive until next spring	summer–next spring [51]	summer–next spring
average time taken until larva moults into nymph*	almost 8 months [51]	275 days
nymph gets blood and then moults into adult	late spring and early summer [51]	late spring and early summer
average time taken until nymph moults into adult*	almost 3–4 months [51]	150 days
eggs → larvae	summer [51]	summer (July)
average time taken until egg hatches into larva*	almost 2–3 months [51]	90 days
adults → time of egg laying	autumn–early spring [52]	autumn–early spring (Dec–May)
average times taken until adult lays eggs*	if fed in autumn, almost 5 months	autumn: 215 days
	if fed in spring, almost immediate [52]	spring: 30 days
female dies after laying egg	1 month [52]	30 days
successful rate of mating for female	autumn: 55.6%–spring: 62.5% [52]	autumn: 0.556
		spring: 0.625
survivability of larva to nymph	unfed: 10.4%–fed: 27% [52,53]	0.16
survival rate of nymph to adult	80.40% [54]	80%
mating rate	male/female = 2.25 [55]	2.25
tick life cycle	2 years [51]	730 days

parameters	estimation interval [reference if any]	calibrated value (confidence interval)
number of eggs per adult female	300–3000 [56,57]	500 (500–510.7)
hatch rate	69% [60.8F]–79% [79F] [53]	different for each walkway
initial number of ticks in each life's stage in each walkway	0–200	different for each walkway
average time of death for larva	150–270 days	150 days (150–153 days)
fraction of birth of mice	4–10 [44]	5.74 (5.41–6)
survival rate of young mice	0.4–0.6	0.54 (0.51–0.57)
probability of death of mice	0.3–0.7	63% (59%–66%)
average infection time	1–2 days [46]	1.99 days (1.7–2 days)
average recovery time after developing LD	4–6 weeks	28 days (28–42 days)
average recovery time after the post-treatment stage	around 10 weeks	80 days (80–200 days)
initial awareness	1%–10%	10% (4%–10%)
desired awareness	30%–50%	30% (30%–31%)
average time to become aware	300–1500 days	300 days (300–595 days)
average time a person passes a walkway	1–4 times	3.87 (3.31–4)

*variables that are defined based on our educated guess to produce a 2-year tick life cycle.

## 3. Results

**Figure 3.**
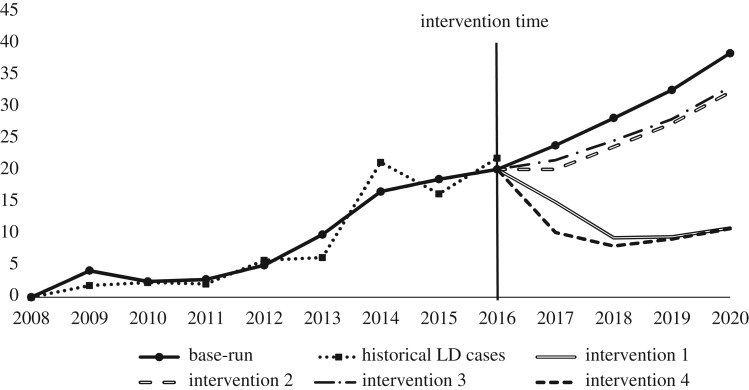
Influence of different interventions on the number of LD Cases.

The final output of the SD model (base-run) in comparison to the New York State Department of Health (NYSDOH) Broome County data series scaled down to the BU Campus (Campus Historical LD cases) between 2008 and 2016 is shown in figure 3. The y-axis shows the LD cases per year and the results of the simulation model matched against the historical trends. If we run the model until year 2020, the model suggests that the number of LD cases will continue to grow if no intervention is applied (see the base-run line in figure 3).

For the purpose of policy design and analysis, we simulated four different intervention scenarios for reducing the number of LD cases between 2016 and 2020 that include (1) doubling awareness of the students from 30% to 60% and consequently their situational awareness through campus media and awareness campaigns over a 12-month period, (2) decreasing the clothing risk of students by 20% in summer and autumn, (3) reducing the population of mice by exterminating them, and (4) increasing efforts to double students' awareness within a 6-month period. As can be seen in figure 3, scenario 4 has the most significant influence on reducing the number of LD cases by 2020, because people become aware in a shorter time period and react to the situation faster. The first scenario has the second best result which supports the importance of raising public awareness and consequently situational awareness. However, reducing the mouse population and decreasing only the clothing risk and not changing situational awareness or preventing other risky behaviours of students did not sufficiently help to decrease the number of LD cases throughout the campus. Thus, in order to effectively prevent the growth of LD cases, educational efforts should be provided to increase public situational awareness to control their risky behaviours for contracting LD.

As an alternative intervention, we also studied the effect of removing nymphs and larvae using our simulation model similar to studies that report controlling hosts for larval and nymph stages (i.e. mainly through birds, mammals and reptiles) that has the strongest effect on controlling tick populations [12]. A series of scenarios are simulated through sensitivity analysis by increasing the probability of death of larva up to 10% and for nymph raised to 80%. The effect of these interventions on the final number of LD cases in the year 2020 is reported in table 4. The results show that eradicating nymphs to 80% has a minor effect on reducing LD cases, but destroying larvae through eradication efforts can decrease LD cases significantly over a 4-year period by 50%. One explanation is that ticks at the larva stage are not yet infected, but at nymphal stage may already be infected. Thus infected ticks at the nymphal stage that survive the eradication might continue spreading LD. However, these interventions need to be validated further and evaluated from an economic efficiency perspective, as to whether it is worth the dollar amount to pursue such efforts.

**Table 4.** Sensitivity analysis scenarios for increasing efforts to eradicate larva and nymph.

percentage increase of probability of death of larva after 2016	cumulative number of LD cases in 2020	percentage increase of probability of death of nymph after 2016	cumulative number of LD cases in 2020
1	203	20	200
4	161	40	197
7	127	60	194
10	106	80	191

## 4. Discussion and conclusion

The number of LD cases has significantly grown over the past 7 years in our region and the northeastern USA (table 2) and our simulation results suggest that if no intervention is applied, LD cases will continue to grow. According to the policy analysis results from our simulation model, in order to effectively prevent the growth of LD cases, educational efforts should be provided to increase public situational awareness so that they can act properly by having the appropriate awareness about their surrounding environment. In other words, solely an increase in public attention does not decrease the risk for LD, but our data suggest the public should maintain a reasonable situational awareness to control their risky behaviours and exposure for contracting LD.

**Figure 4.**
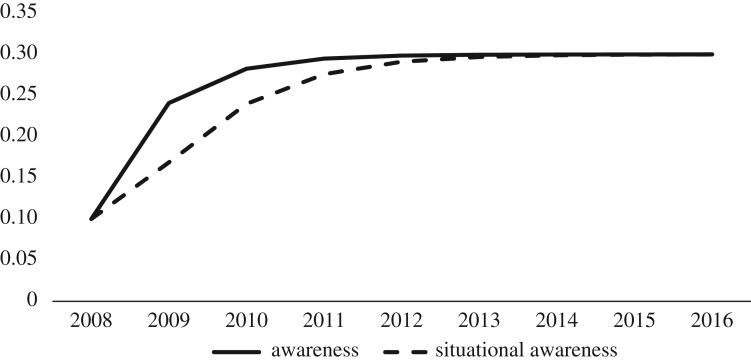
Growth of awareness and situational awareness.

Obviously, if average awareness and situational awareness for LD had reached 100%, then LD risk and consequently the number of LD cases would have dropped significantly. Based on our simulation results, although awareness and situational awareness are increasing over the time period, they just reach 30% by the end of 2013 (see figure 4); hence, the number of LD cases is still growing because a 30% situational awareness by the public is not able to reduce the risk for LD. In other words, people on the University campus do not realize the high risk of contracting LD on campus and do not have sufficient situational awareness about LD. The online University survey we conducted in 2014 and also the demographic and behavioural observational data from 2013 and 2014 that were compiled for different walkways on campus validates these simulation results. Although during the observational and online survey students indicated that they are aware of ticks and of LD, and that they know about the risk of exposure and how they should properly cover their body, in actuality, when they were perambulating along the walkways, they had demonstrated risky behaviours like sitting on the grass, not wearing protective clothing and were not aware of how to check for, recognize and remove ticks. In the online survey, we had 932 respondents, of which 548 were living on campus, and among them, 265 respondents had at least 60% awareness, but 78 of them were bitten by ticks, and 29 of the respondents had at least 35% situational awareness, yet 5 of them still were bitten.

Beaujean *et al*. [26] report results very similar to our findings. The authors ran an online questionnaire with 362 responses. Regarding taking precautions for LD, some major barriers were mentioned including unwillingness to wear protective clothing in the summer due to warm temperatures and ineffectiveness of insect repellent skin products. Hence, the authors concluded that there is a low level of concern about tick bites and LD, and instead of wearing protective clothing, checking skin for ticks is more useful. In addition, some studies found that educational efforts to promote tick checks, avoid tick-infested areas and use repellents for ticks, has a limited impact on changing risky behaviours in order to prevent LD [2]. One study claims that incomplete intervention of repellent use can even increase the disease burden [61]. In other words, only raising awareness through educational efforts is not sufficiently effective in reducing the risk for LD. Jones *et al.* [62] states that increased awareness cannot be interpreted as the execution of preventive measures, and it is much more challenging to change public behaviour and to promote health and LD prevention.

According to the results from our online survey administered in 2014, most of the students claimed that they were aware of ticks and know about the risk of exposure to them. However, during our observational data collection for risky behaviours, there were a high number of people who sat on grass or near low-lying vegetation with bare skin which puts them at a higher risk of exposure to ticks. Although, we would expect that a young age student population may be less cautious about the possible risk of LD, they may also perceive the campus and other peri-urban environments as safe ones that do not expose them to the same level of risk for LD that hiking in the woods or visiting remote wooded parks and other non-built areas might present. This perception leads to low situational awareness and consequently a high risk for LD incidence.

Furthermore, our study demonstrates that environmental factors have a major impact on the risk for LD. According to the data collected on both man-made and organic walkways on the campus, tick density is lower on man-made pavements. However, the simulation results for various walkways show that a significant number of LD cases also occur on man-made walkways, 55 LD cases among all 203 cases. Thus, contrary to suggestions that the risk of tick encounter on pavement areas is small [38], the chance of receiving tick bites on man-made walkways cannot be ignored, even though risk is less when compared with organic walkways. This is often due to the vegetation around the man-made walkways that provide an appropriate environment for questing ticks to attach to low-lying shrubs and tall grass that assists questing ticks to come in contact with people perambulating on these walkways.

The high number of LD cases predicted by our proposed simulation model alludes to the fragmentation effect within the built environment of a university campus that increases the exposure of humans to ticks. In order to stop the increase of tick populations and exposure of humans to them, limitation of forest fragmentation should be considered.

Our system dynamics model provides a simulation framework that could be customized for use in different regions by adjusting the tick and rodent density, tick infectivity, human and environmental risk and awareness levels within that region. Using the simulation results, we can predict the trend for LD cases and awareness growth for the region. The proposed simulation model can be used to prepare a user-friendly web-based platform for public use to assess the individual risk of getting LD. In addition, once this simulation framework is made publicly available, local health departments, as well as state and federal health agencies and other public health organizations, providers, policymakers and the public in general, can use it for any geographical region to have a better understanding of the strength of different interventions on mitigating LD transmission and, therefore, could design more effective interventions and strategies to avoid tick bites. To that end, we are expanding our study to the six-county area that makes up the entire Upper Susquehanna River Basin in New York State.

## Supplementary Material

The Underlying Formula of our System Dynamics Model

## Data accessibility

Data can be found at https://doi.org/10.6084/m9.figshare.5132920.v3.
