# Supplementary material for: Model-based risk assessment and public health analysis to prevent Lyme disease
Source: R Soc Open Sci. 2017 Nov 15;4(11):170841. doi: 10.1098/rsos.170841 (PMC5717649; doi:10.1098/rsos.170841)
Supplement: The Underlying Formula of our System Dynamics Model [file rsos170841supp1.docx]

# Supplementary Materials

Here, we are providing the underlying formula of our system dynamics model. Each module, has its own section.

## Abbreviations and notes:

SA: Situational Awareness

DMNL: Dimensionless

~: explains the units of each variable in our model

SMOOTH, FIXED DELAY: are delay functions in system dynamics modeling

Switch: we can turn on and off this variable

SUM: a function in the software that sums up values over the walkways

Initial Time= 0

Final Time= 4380

~ Day

## Awareness and SA

SA= INTEG (

Increase in SA, Initial SA)

~ Dmnl

Desired SA=

Actual Awareness-Actual SA

~ Dmnl

Increase in SA=

Desired SA/Average Time to Become SA

~ Dmnl/Day

Change in Awareness=

Gap in Awareness/Average Time to Become Aware

~ Dmnl/Day

Gap in Awareness=

Desired Awareness-Actual Awareness

~ Dmnl

Actual Awareness= INTEG (

Change in Awareness, Initial Awareness)

~ Dmnl

## Rodents

Rodent Infection Rate[Walkway]=

Probability of Getting One Bite[Walkway]*(Possible Infected Ticks[Walkway]/Total Tick Population[Walkway])*"Non-Infected Rodents"[Walkway]*(1-Probability of Death for Rodents)/Average Time Delay of Infection for Rodents

~ Rodents/Day

Non-Infected Rodents Dying[Walkway]=

"Non-Infected Rodents"[Walkway]*Probability of Death for Rodents/Average Time of Death for Rodents

~ Rodents/Day

Infected Rodents Dying[Walkway]=

Infected Rodents[Walkway]/Ave Time of Death for Rodents

~ Rodents/Day

Infected Rodents[Walkway]= INTEG (

Rodent Infection Rate[Walkway]-Infected Rodents Dying[Walkway],

Initial Number of Infected Rodents[Walkway])

~ Rodents

"Non-Infected Rodents"[Walkway]= INTEG (

Aging Rate[Walkway]-Rodent Infection[Walkway]-Non-Infected Rodents Dying[Walkway\], Initial Number of Rodents[Walkway])

~ Rodents

Not Young[Walkway]=

Young[Walkway]/Ave Time of Aging of Young Rodents

~ Rodents/Day

Aging Rate of Young Rodents[Walkway]=

Not Young[Walkway]*Survival Rate

~ Rodents/Day

Rodent Birth Rate[Walkway]=

(Infected Rodents[Walkway]+"Non-Infected Rodents"[Walkway])*Fraction of Birth*PULSE TRAIN(\365, 1, 365, 4380)*"Ratio of Female/Male in Rodents"

~ Rodents/Day

Young Rodents Dying[Walkway]=

Not Young[Walkway]*(1-Survival Rate)

~ Rodents/Day

Total Rodent Population[Walkway]=

Infected Rodents[Walkway]+"Non-Infected Rodents"[Walkway]+Young Rodents[Walkway]

~ Rodents

## Ticks

Density of Total Tick Population[Walkway]=

(Total Tick Population[Walkway]/Area of Each Walkway[Walkway])*1000

~ Tick/Thousand m2

"% Infected Blood"[Walkway]=

(Infected Rodents[Walkway]/(Total Rodent Population[Walkway]))

~ Dmnl

Total Tick Population[Walkway]=

Infected Females[Walkway]+Infected Males[Walkway]+Infected Nymphs[Walkway]+Larva[Walkway]+"Non-Infected Adult Females"\[Walkway]+"Non-Infected Adult Males"[Walkway]+"Non-Infected Nymphs"[Walkway]+Mated Females[Walkway]

~ Tick

Add Spring Non Infected[Walkway]= DELAY FIXED (

Switch Spring*Female Mating Rate[Walkway]*Number of Eggs per each Adult female, Ave Delay in Mating Spring\

[Walkway], Switch Spring*Female Mating Rate[Walkway]*Number of Eggs per each Adult female\)

~ Tick/Day

Add Fall Non Infected[Walkway]= DELAY FIXED (

Fall Time*Female Mating Rate[Walkway]*Number of Eggs per each Adult female, Ave Delay in Mating Fall\

[Walkway], Fall Time*Female Mating Rate[Walkway]*Number of Eggs per each Adult female)

~ Tick/Day

Add Spring Infected[Walkway]= DELAY FIXED (

Switch Spring*Infected Female Mating Rate[Walkway]*Number of Eggs per each Adult female\, Ave Delay in Mating Spring[Walkway], Switch Spring*Infected Female Mating Rate[Walkway]\*Number of Eggs per each Adult female)

~ Tick/Day

Total Infected Ticks of all Walkways[Walkway]=

SUM (Possible Infected Ticks[Walkway!])

~ Tick

"Bitten by an Infected Tick (Nymphs or Adults)"[Walkway]=

LD Risk Modeled by Public Awareness[Walkway]*Susceptible Population[Walkway]/Average Infection Time

~ Person/Day

Larva Getting Infected[Walkway]=

Larva Becoming Nymph[Walkway]*(1-"Probability of Death for Larva"[Walkway])*("% Infected Blood"[Walkway\])*Switch Winter*Coefficient

~ Tick/Day

Nymph Growing into Infected Female[Walkway]=

Nymph Becoming Adults[Walkway]*(1-"Probability of Death for Nymphs"[Walkway])*("% Infected Blood"[Walkway\])*(1-Proportion Becoming Male)*Switch Winter*Switch Summer*Coefficient

~ Tick/Day

Nymph Growing into Infected Male[Walkway]=

Nymph Becoming Adults[Walkway]*(1-"Probability of Death for Nymphs"[Walkway])*("% Infected Blood"[Walkway\])*Proportion Becoming Male*Switch Winter*Switch Summer*Coefficient

~ Tick/Day

Risk Factor of Infected Tick Bites[Walkway]=

"Risk of Tick Encounter by Walkway Type - Organic"[Walkway]*"Ratio of Infected Ticks-Total Tick"\[Walkway]

~ Dmnl

Probability of Getting One Bite[Walkway]=

1/(1+EXP(-Steepness[Walkway]*Density of Total Tick Population[Walkway]+Min PGO[Walkway]))

~ Dmnl

~ Logistic Function

"Risk of Tick Encounter by Walkway Type - Organic"[Walkway]=

1/(1+EXP(-Steepness 1[Walkway]*Density of Total Tick Population[Walkway]+YYYY[Walkway]))

~ Dmnl

Density of Infected Ticks[Walkway]=

(Possible Infected Ticks[Walkway]/Area of Each Walkway[Walkway])*1000

~ Tick/Thousand m2

"Nymph -> Adult Transition Rate Female"[Walkway]=

Nymph Becoming Adults[Walkway]*(1-"Probability of Death for Nymphs"[Walkway])*(1-"% Infected Blood"\

[Walkway])*(1-Proportion Becoming Male)*Switch Winter*Switch Summer

~ Tick/Day

"Nymph -> Adult Transition Rate Male"[Walkway]=

Nymph Becoming Adults[Walkway]*(1-"Probability of Death for Nymphs"[Walkway])*(1-"% Infected Blood"\

[Walkway])*Proportion Becoming Male*Switch Winter*Switch Summer

~ Tick/Day

"Larva -> Nymph Transition Rate"[Walkway]=

Larva Becoming Nymph[Walkway]*(1-"Probability of Death for Larva"[Walkway])*(1-"% Infected Blood"[\

Walkway])*Switch Winter

~ Tick/Day

Birth Rate[Walkway]= DELAY FIXED (

(Adults Laying Eggs[Walkway]+Add Fall Non Infected[Walkway]+Add Spring Infected[Walkway]+Add Spring Non Infected\

[Walkway])*Fraction of Become Larva[Walkway], Average Time of Becoming Larva[Walkway], 0)

~ Tick/Day

Female Mating Rate[Walkway]=

(Fall Time *"Non-Infected Adult Females"[Walkway]*(Rate of Mating Fall)*(1-"Probability of Death for Adults"\

[Walkway])/Ave Delay in Mating Fall[Walkway]) + (Switch Spring * "Non-Infected Adult Females"\

[Walkway]*(Rate of Mating Spring)*(1-"Probability of Death for Adults"[Walkway])/Ave Delay in Mating Spring\[Walkway])

~ Tick/Day

"Infected Nymph -> Infected Adult Transtion Rate Female"[Walkway]=

"I-Nymph Becoming Adult"[Walkway]*(1-"Probability of Death for Nymphs"[Walkway])*(1-Proportion Becoming Male\

)*Switch Winter*Switch Summer

~ Tick/Day

Male Mating Rate[Walkway]=

(Fall Time*MIN(Female Mating Rate[Walkway]/"Mating Factor female-male", "Non-Infected Adult Males"\

[Walkway]/Ave Delay in Mating Fall[Walkway])*(1-"Probability of Death for Adults"[Walkway])) + (Switch Spring

*MIN(Female Mating Rate[Walkway]/"Mating Factor female-male", "Non-Infected Adult Males"\

[Walkway]/Ave Delay in Mating Spring[Walkway])*(1-"Probability of Death for Adults"[Walkway]))

~ Tick/Day

"Infected Nymph -> Infected Adult Transtion Rate Male"[Walkway]=

"I-Nymph Becoming Adult"[Walkway]*(1-"Probability of Death for Nymphs"[Walkway])*Proportion Becoming Male\

*Switch Winter*Switch Summer

~ Tick/Day

Infected Female Mating Rate[Walkway]=

(Fall Time * Infected Females[Walkway]*(Rate of Mating Fall)*(1-"Probability of Death for Adults"\

[Walkway])/Ave Delay in Mating Fall[Walkway]) + (Switch Spring * Infected Females[Walkway]*(\

Rate of Mating Spring)*(1-"Probability of Death for Adults"[Walkway])/Ave Delay in Mating Spring\[Walkway])

~ Tick/Day

Infected Male Mating Rate[Walkway]=

(Fall Time*MIN(Infected Female Mating Rate[Walkway]/"Mating Factor female-male", Infected Males\

[Walkway]/Ave Delay in Mating Fall[Walkway])*(1-"Probability of Death for Adults"[Walkway])) + (Switch Spring

*MIN(Infected Female Mating Rate[Walkway]/"Mating Factor female-male", Infected Males[Walkway\

]/Ave Delay in Mating Spring[Walkway])*(1-"Probability of Death for Adults"[Walkway]))

~ Tick/Day

"Non-Infected Adult Females"[Walkway]= INTEG (

("Nymph -> Adult Transtion Rate Female"[Walkway]-"Non-Infected Adults-Female Dying"[Walkway]-Female Mating Rate\

[Walkway]),Initial Non-Infected Adult Female[Walkway])

~ Tick

"Non-Infected Adults-Female Dying"[Walkway]=

"Non-Infected Adult Females"[Walkway]*Switch Killing Adults/TIME STEP

~ Tick/Day

Non-Infected Adults-Males Dying[Walkway]=

"Non-Infected Adult Males"[Walkway]*Switch Killing Adults/TIME STEP

~ Tick/Day

Infected Adults-Male Dying[Walkway]=

Infected Males[Walkway]*Switch Killing Adults/TIME STEP

~ Tick/Day

"Infected Adults-Female Dying"[Walkway]=

Infected Females[Walkway]*Switch Killing Adults/TIME STEP

~ Tick/Day

Infected Nymphs Dying[Walkway]=

Infected Nymphs[Walkway]*Switch Killing Nymphs/TIME STEP

~ Tick/Day

Non-Infected Nymphs Dying[Walkway]=

"Non-Infected Nymphs"[Walkway]*Switch Killing Nymphs/TIME STEP

~ Tick/Day

Infected Females[Walkway]= INTEG (

"Infected Nymph -> Infected Adult Transition Rate Female"[Walkway]+Nymph Growing into Infected Female\

[Walkway]-"Infected Adults-Female Dying"[Walkway]-Infected Female Mating Rate[Walkway], Initial Infected Females[Walkway])

~ Tick

Eggs[Walkway]= INTEG (

Add Fall Non Infected[Walkway]+Add Spring Infected[Walkway]+Add Spring Non Infected[Walkway]+\

Adults Laying Eggs[Walkway]-Birth Rate[Walkway]-Eggs Removal[Walkway],0.001)

~ Tick

Adults Laying Eggs[Walkway]= DELAY FIXED (

Fall Time*Infected Female Mating Rate[Walkway]*Number of Eggs per each Adult female, \

Ave Delay in Mating Fall[Walkway], Fall Time*Infected Female Mating Rate[Walkway]*Number of Eggs per each Adult female\)

~ Tick/Day

Eggs Removal[Walkway]=

Eggs[Walkway]*Switch Killing Eggs/TIME STEP

~ Tick/Day

"Ratio of Infected Ticks/Total Tick"[Walkway]=

Possible Infected Ticks[Walkway]/Total Tick Population[Walkway]

~ Dmnl

"Non-Infected Nymphs"[Walkway]= INTEG (

("Larva -> Nymph Transition Rate"[Walkway]-Non-Infected Nymphs Dying[Walkway]-"Nymph -> Adult Transition Rate Female"\

[Walkway]-"Nymph -> Adult Transtion Rate Male"[Walkway]-Nymph Growing into Infected Female\

[Walkway]-Nymph Growing into Infected Male[Walkway]),0.001)

~ Tick

Larva[Walkway]= INTEG (

Birth Rate[Walkway]-Larva Dying[Walkway]-"Larva -> Nymph Transition Rate"[Walkway]-Larva Getting Infected\[Walkway],

Initial Larva[Walkway])

~ Tick

Mated Females[Walkway]= INTEG (

Female Mating Rate[Walkway]+Infected Female Mating Rate[Walkway]-Dying[Walkway], Initial Mated Females[Walkway])

~ Tick

"Non-Infected Adult Males"[Walkway]= INTEG (

"Nymph -> Adult Transtion Rate Male"[Walkway]-Non-Infected Adults-Males Dying[Walkway]-Male Mating Rate[Walkway],

Initial Non-Infected Adult Males[Walkway])

~ Tick

Infected Males[Walkway]= INTEG (

"Infected Nymph -> Infected Adult Transition Rate Male"[Walkway]+Nymph Growing into Infected Male\

[Walkway]-Infected Adults-Male Dying[Walkway]-Infected Male Mating Rate[Walkway], Initial Infected Males[Walkway])

~ Tick

Infected Nymphs[Walkway]= INTEG (

Larva Getting Infected[Walkway]-Infected Nymphs Dying[Walkway]-"Infected Nymph -> Infected Adult Transition Rate Female"\

[Walkway]-"Infected Nymph -> Infected Adult Transition Rate Male"[Walkway],0.001)

~ Tick

Mated Female Dying[Walkway]=

Mated Females[Walkway]/Average Time of Death for Mated Females[Walkway]

~ Tick/Day

"Infected-Nymph Becoming Adult"[Walkway]=

Infected Nymphs[Walkway]/Ave Time of Becoming Adult[Walkway]

~ Tick/Day

Initial Number of Eggs[Walkway]=SAMPLE IF TRUE(

Time = 1, (Female Mating Rate[Walkway]+Infected Female Mating Rate[Walkway])*Number of Eggs per each Adult female\

*"1 Day", (Female Mating Rate[Walkway]+Infected Female Mating Rate[Walkway])*Number of Eggs per each Adult female\

*"1 Day")

~ Tick

Larva Becoming Nymph[Walkway]=

Larva[Walkway]/Ave Time of Becoming Nymph[Walkway]

~ Tick/Day

Larva Out of eggs[Walkway]=

Eggs[Walkway]/Average Time of Becoming Larva[Walkway]

~ Tick/Day

Possible Infected Ticks[Walkway]=

Infected Nymphs[Walkway]+Total Infected Adult Ticks[Walkway]

~ Tick

Nymph Becoming Adults[Walkway]=

"Non-Infected Nymphs"[Walkway]/Ave Time of Becoming Adult[Walkway]

~ Tick/Day

Total Infected Adult Ticks[Walkway]=

Infected Adult Females[Walkway]+Infected Adult Males[Walkway]

~ Tick

## Human Risk

Overall Human Risk[Walkway]=

Behavioral[Walkway]*Effect of Awareness on LD Risk

~ Dmnl

LD Risk Modeled by Public Awareness[Walkway]=

Overall Human Risk[Walkway]*Risk Factor of Infected Tick Bites[Walkway]

~ Dmnl

"Risky Behavior in Terms of Clothing & Activity"[Walkway]=

GET XLS CONSTANTS( 'Subs.XLS', 'Human Risk', 'B2*' )

~ Dmnl

## SI

Susceptible Population[Walkway]= INTEG (

Recovery Rate from LD[Walkway]+Recovery Rate 2[Walkway]-"Bitten by an Infected Tick (Nymphs or Adults)"\[Walkway],

Number of Times People Pass a Walkway in a Day[Walkway]/Average Time a Person Pass a Walkway\)

~ Person

"Infected Population with (LD)"[Walkway]= INTEG (

"Bitten by an Infected Tick (Nymphs or Adults)"[Walkway]-Developing Post Treatment Lyme Disease\

[Walkway]-Recovery Rate from LD[Walkway],

0)

~ Person

Recovery Rate from LD[Walkway]=

(1-"Fration of Cases Becoming Chronic (Post Treatment Lyme Disease)"[Walkway])*"Infected Population with (LD)"\

[Walkway]/Average Recovery Time After Developing LD

~ Person/Day

Recovery Rate 2[Walkway]=

Post Treatment Lyme Disease[Walkway]/Average Recovery Time After The Post Treatment Stage

~ Person/Day

Post Treatment Lyme Disease[Walkway]= INTEG (

Developing Post Treatment Lyme Disease[Walkway]-Recovery Rate 2[Walkway],0)

~ Person

Developing Post Treatment Lyme Disease[Walkway]=

"Infected Population with (LD)"[Walkway]*"Fraction of Cases Becoming Chronic (Post Treatment Lyme Disease)"\

[Walkway]/Ave Time Developing Post Treatment Lyme Disease[Walkway]

~ Person/Day

## Switches

Summer Time=

IF THEN ELSE((Time>151:AND:Time<=242):OR:(Time>516:AND:Time<=607):OR:(Time>881:AND:Time\

<=972):OR:(Time>1246:AND:Time<=1337):OR:(Time>1611:AND:Time<=1702):OR:(Time>1976:AND:\

Time<=2067):OR:(Time>2341:AND:Time<=2432):OR:(Time>2706:AND:Time<=2797):OR:(Time>3071\

:AND:Time<=3162):OR:(Time>3436:AND:Time<=3527):OR:(Time>3801:AND:Time<=3892):OR:(Time\

>4166:AND:Time<=4257), 1, 0)

~ Dmnl

Switch Not Summer=

IF THEN ELSE((Time>151:AND:Time<=242):OR:(Time>516:AND:Time<=607):OR:(Time>881:AND:Time\

<=972):OR:(Time>1246:AND:Time<=1337):OR:(Time>1611:AND:Time<=1702):OR:(Time>1976:AND:\

Time<=2067):OR:(Time>2341:AND:Time<=2432):OR:(Time>2706:AND:Time<=2797):OR:(Time>3071\

:AND:Time<=3162):OR:(Time>3436:AND:Time<=3527):OR:(Time>3801:AND:Time<=3892):OR:(Time\

>4166:AND:Time<=4257), 0, 1)

~ Dmnl

Winter Time=

IF THEN ELSE((Time>=0:AND:Time<=60):OR:(Time>=333:AND:Time<=425):OR:(Time>=698:AND:Time\

<=790):OR:(Time>=1063:AND:Time<=1155):OR:(Time>=1428:AND:Time<=1520):OR:(Time>=1793\

:AND:Time<=1885):OR:(Time>=2158:AND:Time<=2250):OR:(Time>=2523:AND:Time<=2615):OR:(\

Time>=2888:AND:Time<=2980):OR:(Time>=3253:AND:Time<=3345):OR:(Time>=3618:AND:Time<=\

3710):OR:(Time>=3983:AND:Time<=4075):OR:(Time>=4348:AND:Time<=4440), 1, 0)

~ Dmnl

Switch Not Winter=

IF THEN ELSE((Time>=0:AND:Time<=60):OR:(Time>=333:AND:Time<=425):OR:(Time>=698:AND:Time\

<=790):OR:(Time>=1063:AND:Time<=1155):OR:(Time>=1428:AND:Time<=1520):OR:(Time>=1793\

:AND:Time<=1885):OR:(Time>=2158:AND:Time<=2250):OR:(Time>=2523:AND:Time<=2615):OR:(\

Time>=2888:AND:Time<=2980):OR:(Time>=3253:AND:Time<=3345):OR:(Time>=3618:AND:Time<=\

3710):OR:(Time>=3983:AND:Time<=4075):OR:(Time>=4348:AND:Time<=4440), 0, 1)

~ Dmnl

Switch Killing Eggs=

IF THEN ELSE((Time=273):OR:(Time=642):OR:(Time=1007):OR:(Time=1372):OR:(Time=1737):OR:\

(Time=2102):OR:(Time=2477):OR:(Time=2832):OR:(Time=3197):OR:(Time=3562):OR:(Time=3927\

):OR:(Time=4292), 1, 0)

~ Dmnl

Switch Killing Nymphs=

IF THEN ELSE((Time=333):OR:(Time=698):OR:(Time=1063):OR:(Time=1428):OR:(Time=1793):OR:\

(Time=2158):OR:(Time=2523):OR:(Time=2888):OR:(Time=3253):OR:(Time=3618):OR:(Time=3983\

):OR:(Time=4348), 1, 0)

~ Dmnl

Switch Killing Adults=

IF THEN ELSE((Time=183):OR:(Time=548):OR:(Time=913):OR:(Time=1278):OR:(Time=1643):OR:\

(Time=2008):OR:(Time=2373):OR:(Time=2738):OR:(Time=3103):OR:(Time=3468):OR:(Time=3833\

):OR:(Time=4198), 1, 0)

~ Dmnl

Behavioral[Walkway]=

SMOOTH( ("Risky Behavior in Terms of Clothing & Activity"[Walkway]*(Summer Time+Fall Time)) +

((Winter Time + Switch Spring) * Decrease Clothing Risk*"Risky Behavior in Terms of Clothing & Activity"[Walkway]), "1 month"\)

~ Dmnl

Effect of Awareness on LD Risk=

(Initial X-Min X)*(EXP((-SA)/Beta))+Min X

~ Dmnl

Rate of Mating Fall=

IF THEN ELSE((Time>242:AND:Time<333):OR:(Time>607:AND:Time<698):OR:(Time>972:AND:Time\

<1063):OR:(Time>1337:AND:Time<1428):OR:(Time>1702:AND:Time<1793):OR:(Time>2067:AND:\

Time<2158):OR:(Time>2432:AND:Time<2523):OR:(Time>2797:AND:Time<2888):OR:(Time>3162:AND:\

Time<3253):OR:(Time>3527:AND:Time<3618):OR:(Time>3892:AND:Time<3983):OR:(Time>4257:AND:\

Time<4348), 0.556, 0)

~ Dmnl

Rate of Mating Spring=

IF THEN ELSE((Time>60:AND:Time<=151):OR:(Time>425:AND:Time<=516):OR:(Time>790:AND:Time\

<=881):OR:(Time>1155:AND:Time<=1246):OR:(Time>1520:AND:Time<=1611):OR:(Time>1885:AND:\

Time<=1976):OR:(Time>2250:AND:Time<=2341):OR:(Time>2615:AND:Time<=2706):OR:(Time>2980\

:AND:Time<=3071):OR:(Time>3345:AND:Time<=3436):OR:(Time>3710:AND:Time<=3801):OR:(Time\

>4075:AND:Time<=4166), 0.625, 0)

~ Dmnl
